# Does a poor-quality embryo have an adverse impact on a good-quality embryo when transferred together?

**DOI:** 10.1186/s13048-018-0452-6

**Published:** 2018-09-04

**Authors:** Jiaheng Li, Mingze Du, Zhan Zhang, Yichun Guan, Xingling Wang, Xiao Zhang, Jing Liu, Zhouhui Pan, Bijun Wang, Wenxia Liu

**Affiliations:** 1 The Reproduction Center, The Third Affiliated Hospital of Zhengzhou University, 7 Kangfuqian Road, Zhengzhou, 450052 Henan People’s Republic of China; 2 Waterstone Clinic, Lotamore House, Tivoli, Cork, Ireland

**Keywords:** Embryo quality, Live birth rate, Clinical pregnancy rate, Neonatal outcome

## Abstract

**Background:**

In some in vitro fertilization (IVF) or intracytoplasmic sperm injection (ICSI) cycles, we may consider transferring one poor-quality embryo with one good-quality embryo. Previous studies have indicated that the poor-quality embryo transferred with a good-quality embryo does not negatively affect the clinical pregnancy rate or live birth rate. The purpose of this study was to evaluate pregnancy outcomes and neonatal outcomes in this context.

**Methods:**

This was a retrospective study that included 1646 cycles from our centre. Patients were divided into two groups (group A: one good-quality embryo was transferred with one poor-quality embryo; group B: two good-quality embryos were transferred). The primary outcomes were the clinical pregnancy rate and live birth rate. Additionally, we investigated the implantation rate, ectopic pregnancy rate, abortion rate, multiple pregnancy rate, birthweight and gestational age.

**Results:**

We found that there were no differences in the clinical pregnancy rate and live birth rate between group A and group B. However, the implantation rate and multiple pregnancy rate were higher in group B than in group A.

**Conclusions:**

The poor-quality embryo does not have a significant influence on the good-quality embryo when transferred together.

## Background

The frequency of the use of assisted reproductive technology is increasing. Globally, the number of fresh cycles has increased by 6.4% between 2008 and 2010, and the number of frozen embryo transfer cycles has increased by 27.6% [1]. Double-embryo transfers (DETs) still hold a dominant position despite the increasing number of single-embryo transfer (SET) procedures. In Europe, the rates of SET and DET were 22.4% and 53.2%, respectively, in 2008 and 31.4% and 56.3%, respectively, in 2012 [2, 3]. A meta-analysis found that the live birth rate was lower for SET than for DET, while the live birth rate of a repeated SET (two cycles of SET) was not different from that of a DET [4]. In another meta-analysis, SET reduced the probability of a live birth by 38% and of multiple births by 94%. However, the cumulative live birth rate remained unchanged [5]. Although DET has a higher multiple birth rate than SET, DET also has a higher live birth rate per cycle than SET.

Additionally, the quality of the embryo influences the results of assisted reproductive technology treatment [6–8]. A good-quality embryo is predictive of a better outcome than a poor-quality embryo. However, we cannot guarantee that all embryos are good-quality embryos. What should be done with a poor-quality embryo in assisted reproductive technology treatments? One study indicated that the poor quality of one embryo may influence the quality of another embryo [9]. The blastocyst rate is indeed decreased due to the influence of a poor-quality embryo. Regarding embryo transfer into the uterus, one study pointed out that a poor-quality embryo would affect a good-quality embryo in DET [10]. This study also found that the implantation rates were higher in the control group (transferred two good-quality embryos or one good-quality embryo) than in the study group (transferred one good-quality embryo and one poor-quality embryo). Although previous studies have discussed these problems, the sample sizes have been small. Additionally, previous studies did not discuss the birthweight, gestational age and abortion rate. Therefore, whether we should transfer a poor-quality embryo with a good-quality embryo needed to be evaluated again.

The aim of our research was to determine whether we should transfer a poor-quality embryo with a good-quality embryo.

## Methods

### Study design

This retrospective study was approved by the Ethics Committee of the Third Affiliated Hospital of Zhengzhou University. All patients who underwent in vitro fertilization (IVF) or intracytoplasmic sperm injection (ICSI) at the reproduction centre of the Third Affiliated Hospital of Zhengzhou University between January 2012 and December 2015 were included in this study. Patients who underwent transfer of one or two cleavage-stage embryos were included in our study. We divided these patients into two groups: group A comprised cycles in which one poor-quality embryo was transferred with one good-quality embryo, and group B comprised cycles in which two good-quality embryos were transferred. The outcomes we analysed were the implantation rate, the clinical pregnancy rate, the ectopic pregnancy rate, the abortion rate, the live birth rate, the number of foetuses, birthweight, gestational age, and the incidence of low birth weight and preterm birth.

Clinical pregnancy was diagnosed by transvaginal ultrasound of at least one gestational sac or definitive clinical signs of pregnancy. Live birth was defined as one or more new-borns after 20 completed weeks of gestation. Ectopic pregnancy was defined as a pregnancy that did not occur inside the uterine cavity. Low birth weight was defined as a newborn weight less than 2500 g. Preterm birth was defined as a gestational age of less than 37 weeks.

### Embryo quality

Embryo quality was determined according to a ranking system. Class I embryos were defined as those with six to ten cells on day 3 and < 5% embryo fragmentation. Class II embryos were defined as those with six to ten cells on day 3 and 5–20% embryo fragmentation. Class III embryos were defined as those with uneven cell size, irregular cell morphology, and embryo fragmentation from 21% to 49%. Class IV embryos were defined as those with extremely uneven cell size, a large number of intracellular vacuoles, arrest of embryonic development or embryo fragmentation of over 50%. Class I embryos and Class II embryos were defined as good-quality embryos. Class III embryos were defined as poor-quality embryos, and Class IV embryos were defined as unavailable embryos.

### Inclusion criteria

The inclusion criteria were as follows: fresh IVF/ICSI cycles occurring between January 2012 and December 2015, and patients aged ≤35 years.

### Exclusion criteria

The exclusion criteria were as follows: patients with uterine malformations, patients using donor oocytes, patients who opted for PGD/PGS, and patients with vanishing twins.

### Statistical analysis

The objective of this study was to determine the influence of a poor-quality embryo on a good-quality embryo. The primary outcomes were the clinical pregnancy rate and live birth rate. The secondary outcomes of the study were the implantation rate, the abortion rate, the ectopic pregnancy rate, the multiple pregnancy rate, birthweight and gestational age. Analyses were performed using SPSS (Statistical Package for the Social Sciences) 22.0. We used chi-square tests to evaluate categorical variables. For continuous variables, we used Student’s t-test or the Mann-Whitney test. Logistic regression was performed for live birth and clinical pregnancy using the following variables: maternal age, male age, insemination method, type of infertility, duration of infertility, body mass index, reason for infertility, endometrial thickness and type of cycle. All *p* values less than 0.05 were considered statistically significant.

## Results

We included 1646 cycles in the present study. A total of 1200 cycles involved transfers of two good-quality embryos (group B), and 446 cycles involved transfers of one poor-quality embryo with one good-quality embryo (group A). Body mass index, the reason for infertility, the cause of infertility, male age, endometrial thickness and the insemination method were not significantly different between group A and group B (Table 1). However, we found that male age was lower in group B than in group A (*p* = 0.01). Regarding the outcome of assisted reproductive technology treatment, we found that only the implantation rate was different between group A and group B. Group B had a higher implantation rate (54.8% vs 49.2%, *P* = 0.01) than group A (Table 2). Additionally, group A had a lower multiple pregnancy rate than group B (41.3% vs 33.6%, *P* = 0.02). However, we did not find other differences in the clinical pregnancy rate, the ectopic pregnancy rate, the abortion rate, the live birth rate, birthweight, gestational age, and the incidence of low birth weight or preterm birth. To adjust for confounding factors, we used logistic regression to investigate whether a poor-quality embryo affected a good-quality embryo in terms of the clinical pregnancy rate and live birth rate. We did not observe any differences in the primary outcomes (Table 3).

**Table 1:** Basic information of patients in the two groups

	Group A (*n* = 446)	Group B (*n* = 1200)	*P*
Maternal age (y)	29.0 ± 3.8	28.6 ± 3.2	0.05
Body mass index (kg/m^2^)	22.9 ± 3.3	22.7 ± 3.1	0.17
Duration of infertility (y)	3.5 ± 2.5	3.4 ± 2.5	0.35
Male age (y)	30.5 ± 4.8	29.9 ± 4.6	0.01
Type of infertility			0.47
Primary infertility	266 (59.6%)	692 (57.7%)	
Secondary infertility	180 (40.4%)	508 (42.3%)	
Cause of infertility			0.12
Tubal disease	248 (55.6%)	688 (57.3%)	
Male factor	110 (24.7%)	297 (24.8%)	
Endometriosis	62 (13.9%)	176 (14.7%)	
Unexplained	26 (5.8%)	39 (3.3%)	
Endometrial thickness (mm)	11.4 ± 2.3	11.3 ± 2.2	0.33
Insemination method			0.81
IVF	326 (73.1%)	870 (72.5%)	
ICSI	120 (26.9%)	330 (27.5%)

**Table 2:** Clinical and neonatal outcomes in the two groups

	Group A (*n* = 446)	Group B (*n* = 1200)	*P*
Implantation rate	49.2% (439/892)	54.8% (1314/2400)	0.01
Clinical pregnancy rate	71.3% (318/446)	74.7% (896/1200)	0.17
Ectopic pregnancy rate	1.3% (4/318)	2.7% (24/896)	0.15
Abortion rate	8.8% (28/318)	9.7% (87/896)	0.64
Live birth rate	64.1% (286/446)	65.4% (785/1200)	0.63
Number of foetuses			0.02
Singleton	190 (66.4%)	461 (58.7%)	
Twin	96 (33.6%)	324 (41.3%)	
Birthweight			
Singleton	3346 ± 545	3318 ± 563	0.67
Twin	2596 ± 402	2567 ± 436	0.40
Low birth weight			
Singleton	4.2% (8/190)	4.8% (22/461)	0.76
Twin	35.4% (68/192)	39.0% (253/648)	0.36
Gestational age			
Singleton	38.8 ± 1.5	38.7 ± 1.8	0.42
Twin	36.5 ± 1.9	36.4 ± 1.9	0.86
Preterm birth			
Singleton	7.9% (15/190)	5.9% (27/461)	0.34
Twin	39.6% (38/96)	36.4% (118/324)	0.57

**Table 3:** Logistic regression of clinical pregnancy and live births

Predictors	AOR^a^ (95%CI)	*P*
Clinical pregnancy		
Maternal age	0.959 (0.922–0.996)	0.032
Body mass index	1.031 (0.995–1.069)	0.094
Duration of infertility	0.886 (0.685–1.147)	0.359
Type of infertility	0.952 (0.750–1.208)	0.686
Male age	0.991 (0.957–1.026)	0.612
Endometrial thickness	1.098 (1.044–1.155)	< 0.001
Group C	0.839 (0.655–1.076)	0.166
Live birth		
Maternal age	0.936 (0.895–0.980)	0.005
Body mass index	1.003 (0.971–1.037)	0.843
Duration of infertility	1.065 (0.838–1.355)	0.605
Type of infertility	1.067 (0.856–1.329)	0.563
Male age	1.008 (0.976–1.042)	0.615
Endometrial thickness	1.096 (1.046–1.148)	< 0.001
Group	0.952 (0.755–1.200)	0.676

*AOR* adjusted odds ratios

^a^Adjusted for maternal age, male age, insemination method, type of infertility, duration of infertility, body mass index, reason for infertility and endometrial thickness

## Discussion

A poor-quality embryo can provide a chance to achieve pregnancy. However, a previous study suggested that transfer of a poor-quality embryo would lead to a reduction in the clinical pregnancy rate and live birth rate. However, there were no differences between transfer of a poor-quality embryo and transfer of a good-quality embryo in terms of the abortion rate, pregnancy complications, maternal complications or neonatal complications [6]. In another study, authors found that a poor-quality embryo may affect a good-quality embryo in vitro [9]. Therefore, we wondered whether a poor-quality embryo had an adverse influence on a good-quality embryo. Two experts have previously discussed this topic. One expert suggested that the poor-quality embryo would influence the pregnancy rate and implantation rate [11], and the other expert indicated that transfer of one poor-quality embryo with one good-quality embryo resulted in a lower implantation rate than transfer of two good-quality embryos [10]. However, those researchers did not find a difference in the clinical pregnancy rate, live birth rate, abortion rate, ectopic pregnancy rate or multiple pregnancy rate.

Compared with groups that underwent transfer of one good-quality embryo, group A and group B had higher clinical pregnancy rates and live birth rates. However, we did not observe differences between group A and group B. Hence, the poor-quality embryo may not affect these outcomes. After adjusting for confounding factors, we did not find that the conditions of the two groups had any influence on the outcomes. Consistent with a previous article, we did not find evidence that the poor-quality embryo affected the live birth rate or pregnancy rate. Additionally, we found that the implantation rate was lower in group A than in group B, although two previous articles had indicated that good-quality embryos were easier to implant and develop than poor-quality embryos. However, we found that the multiple pregnancy rate was higher in group B. In other studies, the multiple pregnancy rate was not significantly different between patients who underwent different embryo transfer schemes. We hypothesized that a high implantation rate was related to a high multiple pregnancy rate. After all, a good-quality embryo has a higher chance of implantation and development than a poor-quality embryo [7, 8]. Regarding the abortion rate and ectopic rate, there were no differences between the two groups. We also specifically investigated the birthweight, gestational age, and incidence of low birth weight and preterm birth. However, after dividing pregnancies into singletons and twins, we found no differences in these outcomes. Hence, the poor-quality embryo did not influence birthweight or gestational age.

In summary, we found that only the implantation rate and multiple pregnancy rate were different between group A and group B. We did not find significant differences in the other parameters. A high multiple pregnancy rate is associated with high risks of infant death, low birth weight, deformational plagiocephaly, and other problems [12]. To reduce the risk of a multiple pregnancy, we should consider the conditions of group A as an alternative transfer scheme with no difference in the live birth rate compared to that in group B.

The clinical pregnancy rate (74.7% vs 71.3%, *P* = 0.17) and live birth rate (65.4% vs 64.1%, *P* = 0.63) were higher in group B than in group A; however, the difference was not statistically significant. We tried our best to reduce the influence of confounding factors by optimizing the inclusion and exclusion criteria. However, our study has several limitations. First, this study was a retrospective study, and perhaps a randomized controlled trial would be more persuasive. Second, the criteria for the evaluation of embryo quality were subjective. Finally, additional big-data research studies and a systematic review are required to confirm these findings.

## Conclusion

The poor-quality embryo does not significantly influence the good-quality embryo when transferred together.

## Abbreviations

DET: Double-embryo transfer
ICSI: Intracytoplasmic sperm injection
IVF: In vitro fertilization
SET: Single-embryo transfer
